# Disentangling the Relative Roles of Vertical Transmission, Subsequent Colonizations, and Diet on Cockroach Microbiome Assembly

**DOI:** 10.1128/mSphere.01023-20

**Published:** 2021-01-06

**Authors:** Justinn Renelies-Hamilton, Kristjan Germer, David Sillam-Dussès, Kasun H. Bodawatta, Michael Poulsen

**Affiliations:** a Section for Ecology and Evolution, Department of Biology, University of Copenhagen, Copenhagen, Denmark; b Université Sorbonne Paris Nord, Laboratoire d’Ethologie Expérimentale et Comparée UR4443, Villetaneuse, France; c Natural History Museum of Denmark, University of Copenhagen, Copenhagen, Denmark; University of California, Davis

**Keywords:** host specificity, microbial inocula, microbiome stability, network analysis, symbiosis, transmission

## Abstract

A multitude of factors affect the assemblies of complex microbial communities associated with animal hosts, with implications for community flexibility, resilience, and long-term stability; however, their relative effects have rarely been deduced. Here, we use a tractable lab model to quantify the relative and combined effects of parental transmission (egg case microbiome present/reduced), gut inocula (cockroach versus termite gut provisioned), and varying diets (matched or unmatched with gut inoculum source) on gut microbiota structure of hatchlings of the omnivorous cockroach Shelfordella lateralis using 16S rRNA gene (rDNA) amplicon sequencing. We show that the presence of a preexisting bacterial community via vertical transmission of microbes on egg cases reduces subsequent microbial invasion, suggesting priority effects that allow initial colonizers to take a strong hold and which stabilize the microbiome. However, subsequent inoculation sources more strongly affect ultimate community composition and their ecological networks, with distinct host-taxon-of-origin effects on which bacteria establish. While this is so, communities respond flexibly to specific diets in ways that consequently impact predicted community functions. In conclusion, our findings suggest that inoculations drive communities toward different stable states depending on colonization and extinction events, through ecological host-microbe relations and interactions with other gut bacteria, while diet in parallel shapes the functional capabilities of these microbiomes. These effects may lead to consistent microbial communities that maximize the extended phenotype that the microbiota provides the host, particularly if microbes spend most of their lives in host-associated environments.

**IMPORTANCE** When host fitness is dependent on gut microbiota, microbial community flexibility and reproducibility enhance host fitness by allowing fine-tuned environmental tracking and sufficient stability for host traits to evolve. Our findings lend support to the importance of vertically transmitted early-life microbiota as stabilizers, through interactions with potential colonizers, which may contribute to ensuring that the microbiota aligns within host fitness-enhancing parameters. Subsequent colonizations are driven by microbial composition of the sources available, and we confirm that host-taxon-of-origin affects stable subsequent communities, while communities at the same time retain sufficient flexibility to shift in response to available diets. Microbiome structure is thus the result of the relative impact and combined effects of inocula and fluctuations driven by environment-specific microbial sources and digestive needs. These affect short-term community structure on an ecological time scale but could ultimately shape host species specificities in microbiomes across evolutionary time, if environmental conditions prevail.

## Abstract (precis)

When host fitness is dependent on gut microbiota, microbial community flexibility and reproducibility enhance host fitness by allowing fine-tuned environmental tracking and sufficient stability for host traits to evolve. Our findings lend support to the importance of vertically transmitted early-life microbiota as stabilizers through interactions with potential colonizers that may contribute to ensuring that the microbiota aligns within host fitness-enhancing parameters.

## INTRODUCTION

Intricate associations between animal hosts and their gut microbiota are vital for the evolution and persistence of animal hosts (1, 2). These microbial symbionts facilitate a multitude of functions associated with host nutrient management, immunity, and development (1, 3) and ultimately impact host adaptation and diversification across environments and dietary niches (4–8). When host traits select for specific microbial functions, these can be considered the extended phenotype of the host (9–12). Thus, selection should optimally lead to a microbiota that is both functionally flexible (i.e., able to incorporate environment-specific strains that are likely to enable degradation of environment-specific nutrients and toxins) and functionally consistent (i.e., similar under a defined set of circumstances, leading to a reproducible microbiome assembly) rather than be subject to random fluctuations (3, 13–15). Along those lines, the Anna Karenina principle states that healthy microbiomes are more similar to each other than microbiomes under a range of perturbations (16, 17), a paradigm we will use to understand microbiome assembly reproducibility. Microbiome flexibility and consistency can be achieved either through having a taxonomically fixed microbial community with high functional diversity (18, 19) or by shifts in taxon compositions to accommodate functional needs (13, 20, 21).

The composition of complex gut microbial communities in many mammals, birds, and insects (22) is driven by host taxonomy (23–26), diet (4, 5), vertical (parent-offspring) transmission (27), and environmental inputs (28), including transmission through social behaviors (29) (e.g., coprophagy [30, 31] and trophallaxis [30]). Early-life microbial colonizations, including vertical transmission and environmental inputs, will have a disproportionate effect on the microbiota (priority effects), with subsequent positive (facilitation) and negative (competition) interactions between community members affecting ultimate composition (32–35). In addition, gut physiology and diet impose strong filters that limit what microbes can establish and ultimately diversify with host species (34, 36, 37). Diets will on average be more similar between individuals of the same host species than between species, and they may hence contribute to microbiota consistency within species on ecological time scales (38) and ultimately long-term association across evolutionary time (2). Studies exploring the impact of host phylogeny (e.g., reference 39), diet (39, 40), or microbial inocula (36) on microbiota structure show that each of these has considerable impact on structure and composition of microbiome, yet few have tested their relative and combined effects.

To contribute to closing this knowledge gap, we quantify the relative and combined effects of transmission (with or without disrupted vertical transmission), environmental microbial sources, various diets, and host specificity on gut microbiota structure in hatchlings of the omnivorous cockroach Shelfordella lateralis (Turkestan cockroach; Blattodea: Blattidae). Cockroaches are excellent models for testing this, as their microbiomes are diverse yet consistent within a cockroach species, while being amenable to antimicrobial and dietary manipulations (40–43). We exposed developing nymphs with or without access to bacterial communities on the ootheca (the egg cases they emerge from, a potential source of parental gut microbes), as whether the importance of this indirect vertical transmission route is similar to that in other cockroaches remains unknown (44, 45). Next, we exposed both groups of nymphs to microbial inocula from their own species (conspecific) or from another species (allospecific) (cockroach versus termite) and the corresponding diets of the two species (omnivorous versus specialized fungus). We chose these species because termites are social cockroaches and fungus-growing termites share many cockroach gut bacterial lineages (23, 46). In doing so, we show that ecological interactions are important for microbial assemblage and that removing the initial microbiome, even if later reinoculated, will strongly affect microbiome consistency, even if most microbes are shared. Subsequent differences in available microbial inoculum sources and diets, in combination, drive ultimate microbiota structure and predicted function.

## RESULTS

### Antimicrobial treatment effectively reduces host gut microbiome.

We confirmed that antimicrobial treatment with peracetic acid of ootheca reduced bacterial diversity in the initial microbiota of developing *S. lateralis* nymphs, using an established protocol that does not appear to negatively affect the cockroaches (37, 47). First, we compared the number of bacterial CFU (see Fig. S1 at https://www.doi.org/10.5281/zenodo.4074900) that emerged on growth medium after plating of cockroaches from treated/untreated ootheca and found that the number was significantly lower in treated individuals (mean CFU/sample ± SE: 5.08 ± 2.48 and 55.33 ± 20.34, respectively; Welch approximate degrees of freedom [WelchADF]; WJ = 5.781, df = 1, *P* = 0.0434; see Fig. S1 at the Zenodo URL above). Second, we performed HiSeq amplicon sequencing of one cockroach per treated or untreated ootheca used in the main experiment (see below) and found a reduction in amplicon sequence variant (ASV) diversity by 36.8% in nymphs from treated ootheca (Kruskal-Wallis; χ^2^ = 4.333, df = 1, *P* = 0.0374; see Table S1 at the Zenodo URL above), but no effect of treatment on community richness (χ^2^ = 2.077, df = 1, *P* = 0.1495) or beta diversity (PERMANOVA_10,000 permutations_; F_1_ = 1.268, *P* = 0.2873). Overlap between control 1-day-old and 33-day-old nymphs included 7 genera absent in antimicrobial-treated 1-day-old nymphs (see Fig. S2 at the Zenodo URL above). As expected, the obligate endosymbiont *Blattabacterium* was unaffected by treatment (cf. reference 48), which was interestingly also the case for an *Alistipes*_III ASV (see Fig. S3 at the Zenodo URL above), suggesting that it is either resistant to peracetic acid or vertically transmitted within the ootheca and hence not exposed to the treatment. All other ASVs were reduced in relative abundance, often below the level of detection (see Fig. S3 at the Zenodo URL above). Had we been able to fully remove all bacteria in the peracetic acid treatment, we predict that it would only have exacerbated our results, as most of the bacteria that remained overlap between treated and control cockroaches (see Fig. S2 and S3 at the Zenodo URL above). At the same time, six core genera were detected in all experimental groups, including 1-day-old treated and control nymphs (see Fig. S2 at the Zenodo URL above). However, in summary, although treatment with peracetic acid did not sterilize developing nymphs, microbiome diversity was substantially depleted.

### The combined effects of antimicrobial treatment, inoculum, and diet on microbiome structure.

**FIG 1:**
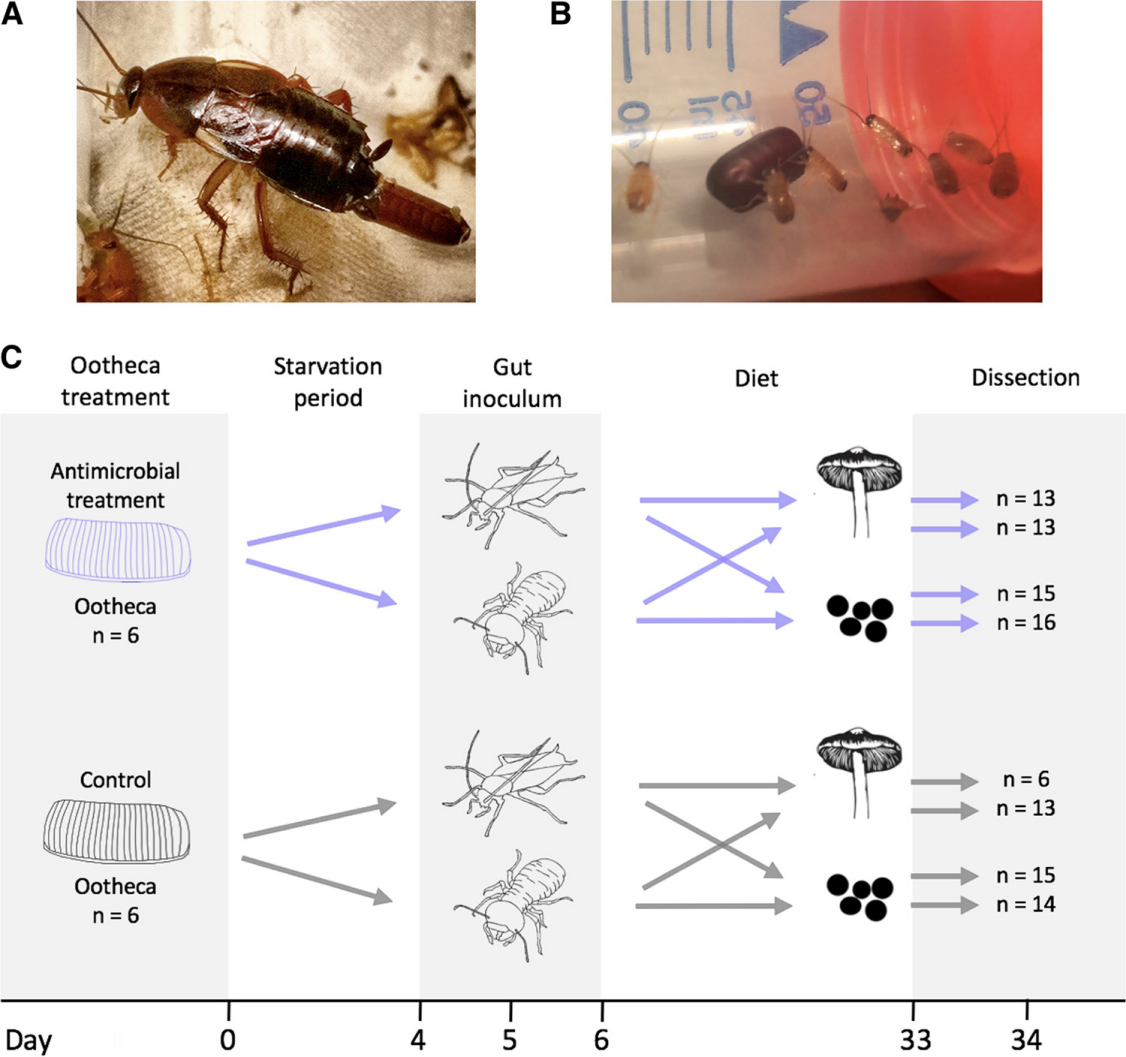
(A) *Shelfordella lateralis* laying an ootheca with eggs. (B) Newly hatched *S. lateralis* nymphs with their ootheca (photos by K.G.). (C) Schematic of the main experimental setup and timeline; silhouette of termites and mushrooms by Rafael R. da Costa (courtesy of Rafael R. da Costa, reproduced with permission), silhouette of cockroach and dog food by K.H.B. Cockroaches hatched on day 0, and a single cockroach was removed to assess antimicrobial treatment effectiveness on day 1. Blue arrows represent the oothecae treated with peracetic acid to deplete the microbiome, and gray arrows represent controls. Sample sizes are indicated with “n.”

Using 16S rRNA gene (rDNA) HiSeq amplicon sequencing of whole guts after a 28-day treatment period, we tested for the combined effects of the depletion of the microbiome on the ootheca (presence/absence of peracetic acid treatment), environmental inocula (*S. lateralis* versus Macrotermes subhyalinus gut provided on the first day of the experiment), genetic background (ootheca identity), and diet (omnivorous dog food versus specialized fungus diet provided *ad libitum*) (Fig. 1 shows the experimental setup, and Materials and Methods provides details). We did observe overall more mortality among cockroaches on a fungal diet (see Fig. S4 at https://www.doi.org/10.5281/zenodo.4074900), indicating that the dog food diet might be better for the cockroaches, possibly because they are not adapted to a fungal diet. Notably, this effect is rescued when inoculated with the termite microbiome, which is better at degrading fungal biomass (see below), and presumably contributes to energy uptake from this diet. However, seven of our eight treatment groups (i.e., diet-inoculum combinations) were not significantly different from each other (see Fig. S4 and Table S2 at the Zenodo URL above), suggesting no overall impact on the experiment or on the conclusions we derive.

**FIG 2:**
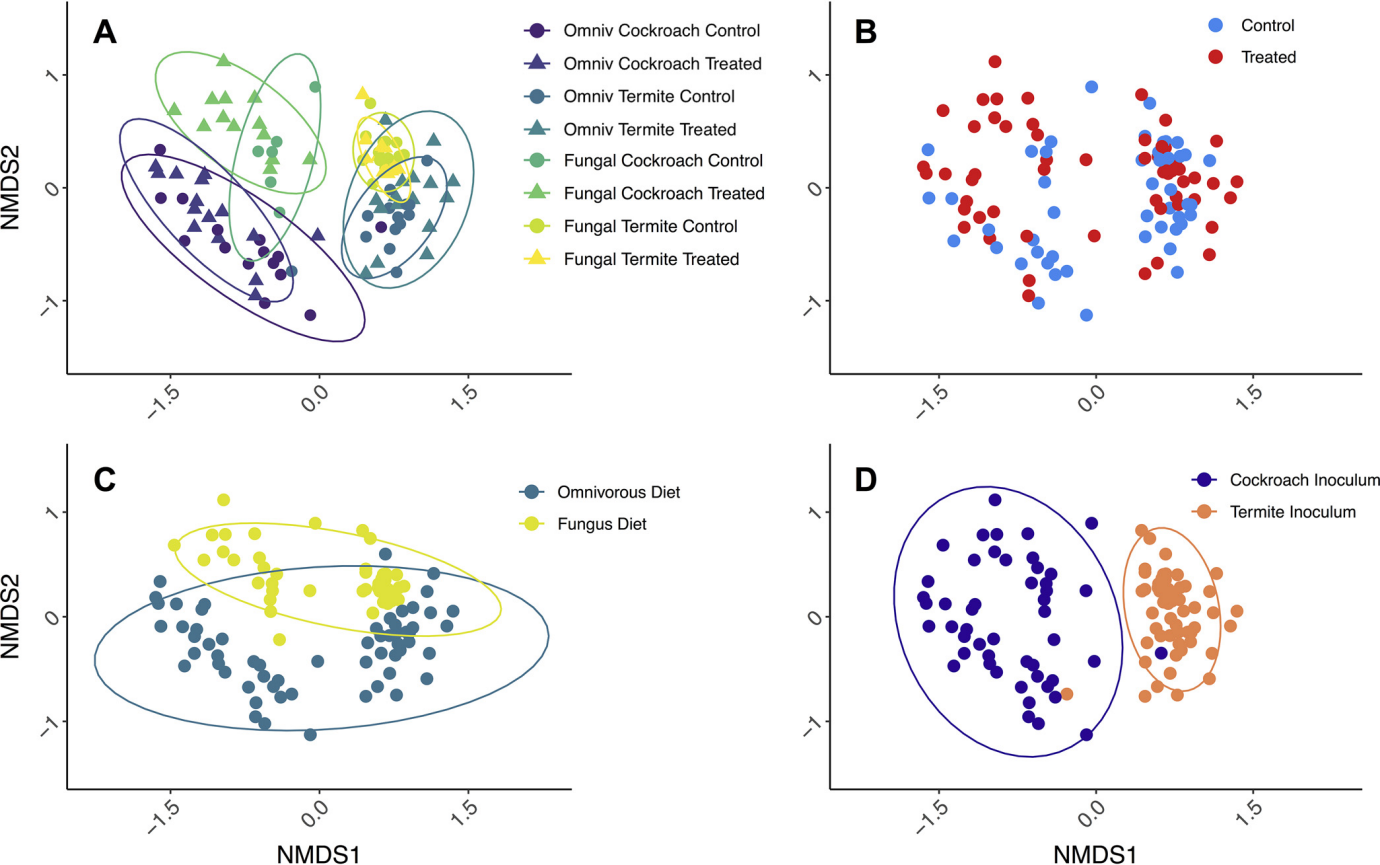
Bray-Curtis distance NMDS ordination plot with identical coordinates in all panels, stress = 18.2%. In panel A, triangles indicate peracetic acid-treated cockroaches and circles indicate controls. Color indicates treatment groups: cockroach gut and omnivorous diet in purple, termite guts and omnivorous diet in dark green, cockroach guts and fungus diet in teal, and termite guts and fungus diet in yellow (Fig. 3). Panel B is colored by antimicrobial treatment (treatment in red and control in blue), panel C is colored by diet (omnivorous in blue and fungal in ocher), and panel D is colored by microbial inoculum (cockroach gut in purple and termite gut in orange). Ellipses indicate 95% confidence intervals.

Ordination analyses (Fig. 2) revealed grouping in nonmetric multidimensional scaling (NMDS) space that were consistent with strong effects of inoculum (PERMANOVA_10,000 permutations_; F_1_ = 15.24, *P* < 0.0001, *R*^2^ = 0.1145) (Fig. 2D), diet (F_1_ = 8.734, *P* < 0.0001, *R*^2^ = 0.0657) (Fig. 2C), and minor effects of antimicrobial treatment (F_1_ = 3.422, *P* < 0.0001, *R*^2^ = 0.0257) (Fig. 2B). Although the analyses indicated that host genetic background (ootheca origin) was significant (F_10_ = 1.561, *P* < 0.0001, *R*^2^ = 0.1174), samples did not cluster accordingly in the NMDS plot (see Fig. S5 at https://www.doi.org/10.5281/zenodo.4074900), suggesting that this result is influenced by the number of groups this factor contained, and that it hence should be interpreted with caution (see Table S3 at the Zenodo URL above). The patterns observed in the NMDS analysis presented in Fig. 2 are robust to alternative beta diversity measures (unweighted Bray-Curtis [see Fig. S6 at Zenodo URL above] and UniFrac [see Fig. S7 and S8 at Zenodo URL above] distances) and analytical methods (principal-coordinate analysis [PCoA]; see Fig. S6, S8, and S9 at Zenodo URL above). The outcome of the beta diversity measures also did not differ between the original and the rarefied data set (see Table S4 at Zenodo URL above).

**FIG 3:**
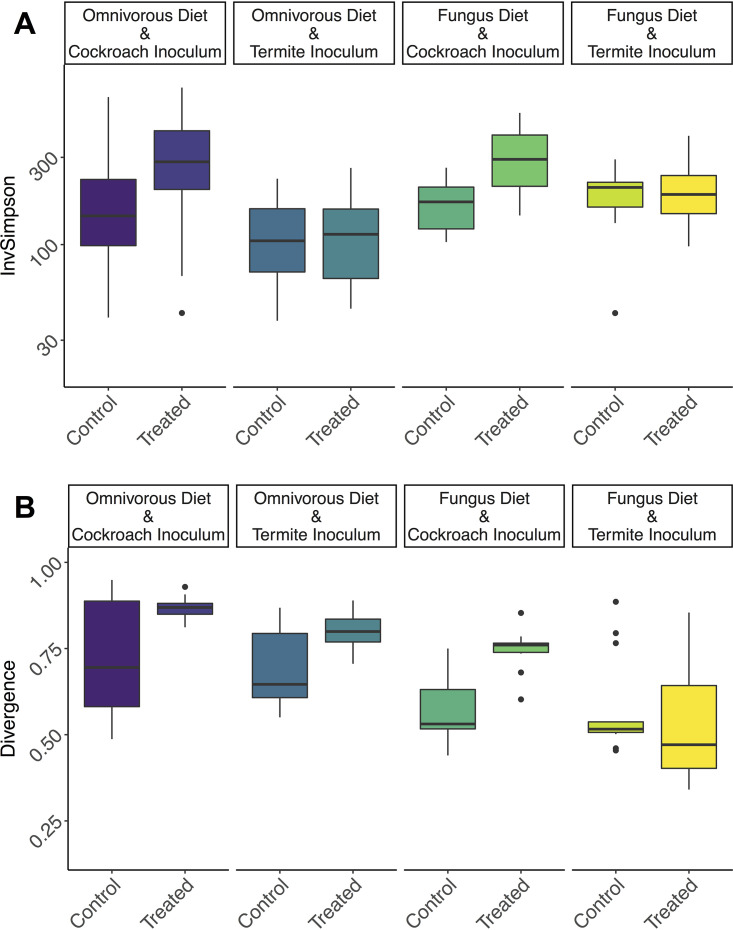
(A) Inverse Simpson diversity index for each treatment group on a log_10_ scale. (B) Microbiome divergence (inverse metric for consistency) calculated as beta diversity between each sample in a group and the representative median abundance of each microbe in that group. Horizontal lines indicate medians, and hinges indicate first and third quantiles. Coloration of treatment groups corresponds to that of Fig. 2A.

Gut microbiome alpha diversity was affected by diet, inoculum, and antimicrobial treatment but unaffected by cockroach genetic background (Fig. 3A; also see Fig. S10 and Table S5 at https://www.doi.org/10.5281/zenodo.4074900). Antimicrobial-treated cockroaches regain a more diverse gut microbiome following microbial inoculation, especially when inoculum is sourced from conspecifics (analysis of variance [ANOVA]; F_1_ = 6.522, adjusted *P* [p_adj_] = 0.0122) (Fig. 3A). Cockroach inocula produced on average 69.7% more diverse communities than termite inocula, indicating that conspecific inoculum allows more microbial gut colonization (F_1_ = 17.12, p_adj_ = 8e−5). Conversely, however, the fungal diet sustained on average 17.7% more diverse communities than the omnivorous diet that the cockroaches are normally raised on (F_1_ = 8.547, p_adj_ = 0.0044). Identical patterns were detected after rarefaction (see Table S6 at the Zenodo URL above), so we provide only the results of the original data set. In line with the beta diversity results, inoculum had the largest effect size (0.4701), followed by diet (0.3366) and antimicrobial treatment (0.2918) (see Table S5 at the Zenodo URL above).

To explore whether disrupting the early-life microbiome and varying microbial inocula and diets affected how consistent microbiomes were within treatments, we modeled between-sample divergence within groups (see Materials and Methods) (49). Antimicrobial treatment increased microbiome variability (and hence, decreased microbiome consistency) (WelchADF; WJ = 16.81, df = 1, *P* = 0.0002) (Fig. 3B; see also Table S7 at https://www.doi.org/10.5281/zenodo.4074900), while inoculation with cockroach gut bacteria increased variability compared to termite guts (WJ = 8.905, df = 1, *P* = 0.0046), as did an omnivorous compared to a fungal diet (WJ = 45.075, df = 1, *P* = 2.672e−8). Lastly, there was a significant compensatory or negative interaction between inoculum and antimicrobial treatment. Antimicrobial-treated cockroaches fed on cockroach inocula were significantly more variable than controls, and antimicrobial-treated individuals inoculated with a cockroach microbiome experienced greater variability than did individuals inoculated with termite microbes (WJ = 7.048, df = 1, *P* = 0.0109).

### Taxon effects driving the community-level differences associated with treatment.

**FIG 4:**
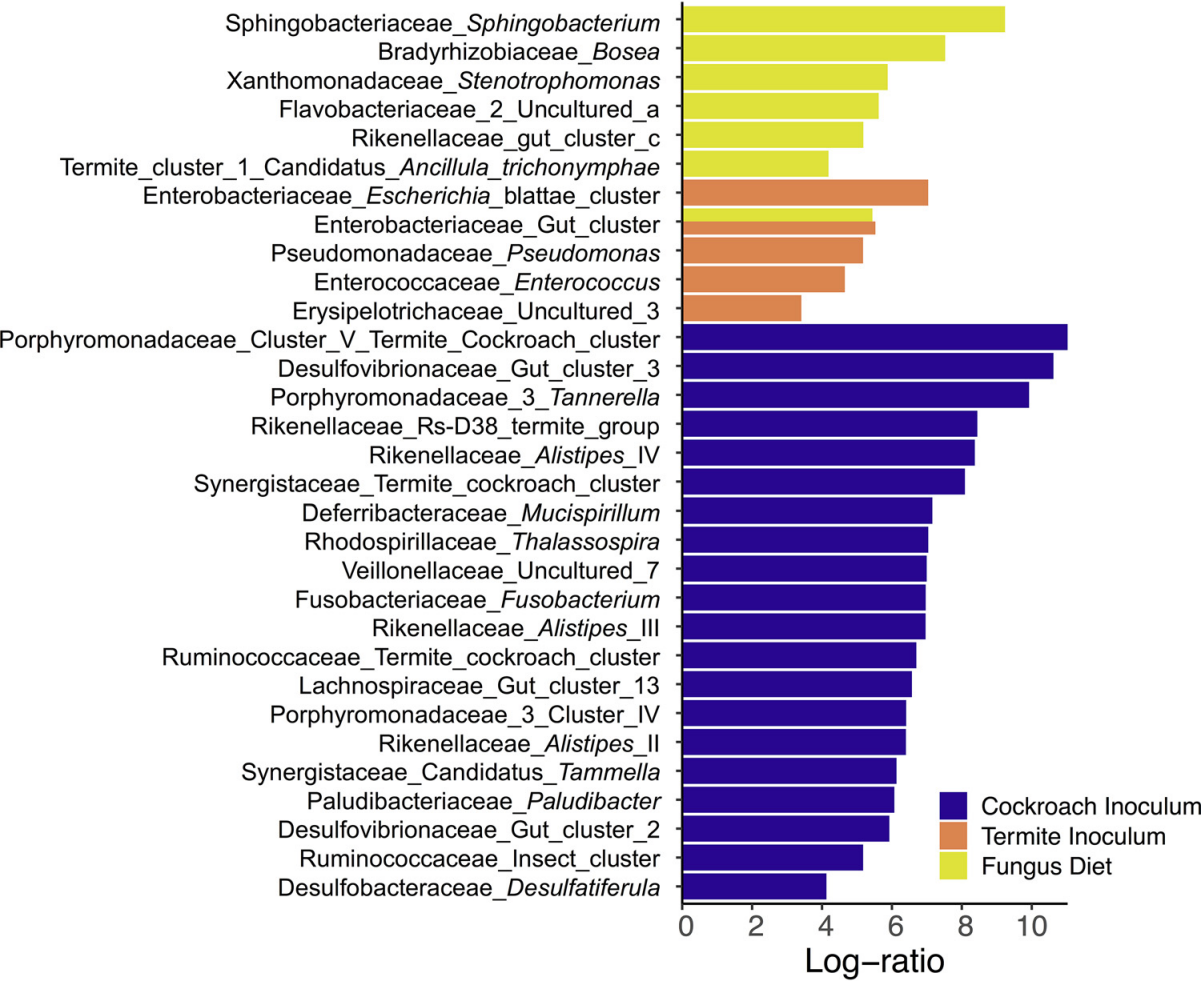
Log-ratio (effect size) increase in genera significantly correlating with inoculum or diet compared to their alternative. Calculated with a multivariate Aldex2 generalized linear mixed model. All plotted taxa have Bonferroni-Hochberg-adjusted *P* values below 1e−5.

To determine what taxa were affected most by our treatments, we used an ANOVA-like multivariate model (Aldex2) (17, 50, 51) while accounting for the independent effects of antimicrobial treatment, diet, and inoculum. Twenty genera increased significantly in relative abundance in groups receiving a cockroach inoculum, five genera increased in groups with a termite inoculum, and seven genera increased in cockroaches on a fungus diet (Fig. 4; see also Table S8 at https://www.doi.org/10.5281/zenodo.4074900). Interestingly, no taxa increased significantly in relative abundance in cockroaches fed the omnivorous diet. The set of taxa that increased in abundance with the cockroach inoculum included Porphyromonadaceae_Cluster_V termite_cockroach_cluster; *Alistipes_*II, III, and IV; Ruminococcaceae insect_cluster and termite_cockroach_cluster; *Mucispirillum*; Veillonellaceae uncultured_7, Lachnospiraceae gut_cluster_13; Desulfovibrionaceae Gut_cluster_II; and Synergistaceae termite_cockroach_cluster (Fig. 4). All of these taxa have diversified within the Blattodea to some extent and are found in both fungus-growing termites and cockroaches (36, 52).

**FIG 5:**
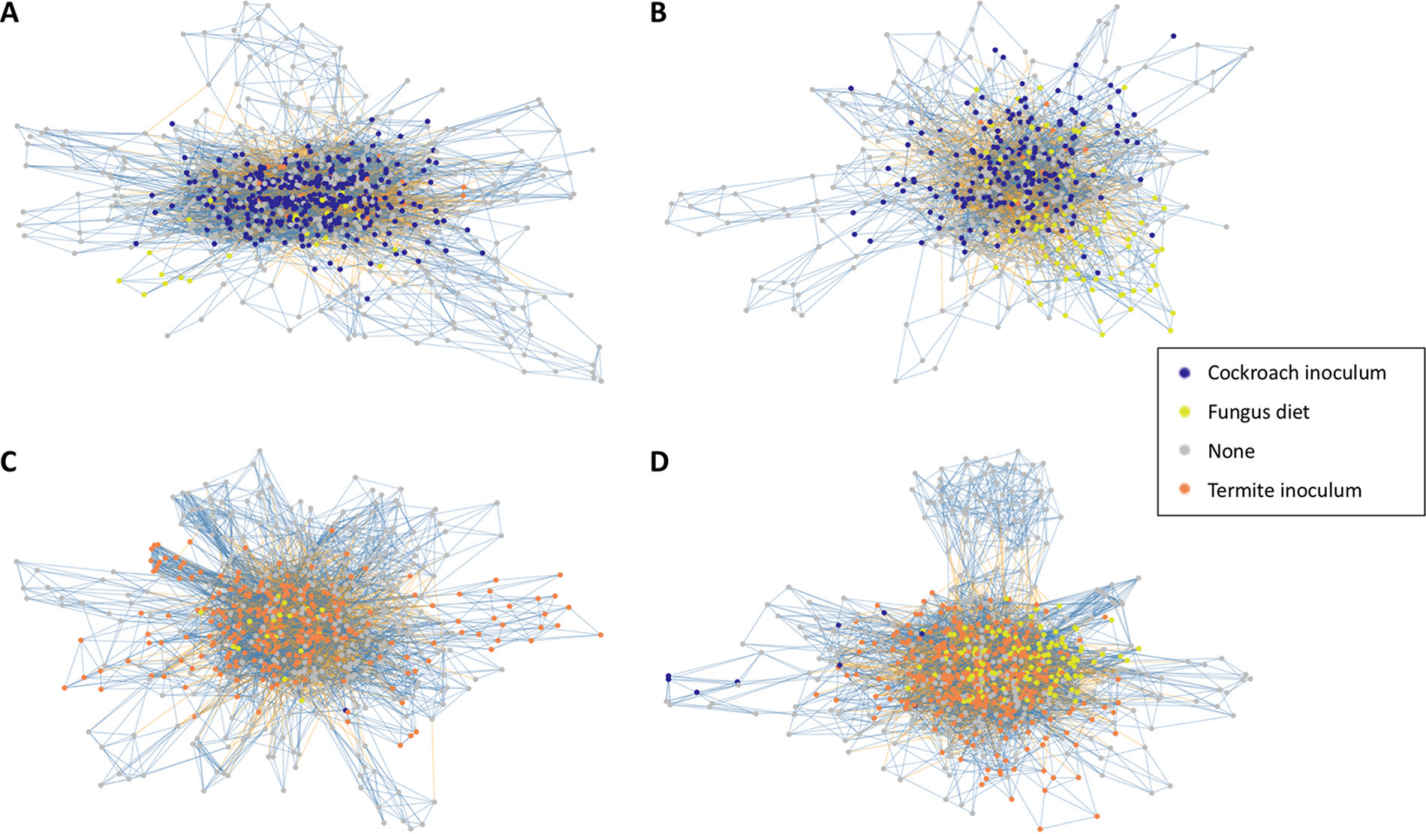
ASV-level networks, where each node represents an ASV, and if that ASV belongs to a differentially abundant genus, it is colored (Fig. 4). Vertices are colored by correlation direction (blue, positive; yellow, negative). (A) Cockroach inoculum and omnivorous diet; (B) cockroach inoculum and fungus diet; (C) termite inoculum and omnivorous diet; (D) termite inoculum and fungus diet.

To explore connections between taxa within the communities, we performed network analyses independently for each diet-inoculum treatment (Fig. 5). These networks were complex, but some patterns emerged. Most interestingly, treatments with matched diets and microbiota exhibited many more interactions (>6,000) than mismatched combinations: 4,701 for cockroaches with termite guts on an omnivorous diet and 2,976 for cockroaches with cockroach guts and a fungal diet (see Table S9 at https://www.doi.org/10.5281/zenodo.4074900). Notably, the former two also had the largest number of taxa, but even after correcting for this, cockroaches with termite guts on an omnivorous diet still had the most connections per taxon (see Table S9 at the Zenodo URL above). Although connections were fewer, both groups with omnivorous diets had a larger proportion of positive interactions (69.1 to 70.2%), while these were slightly lower (65.5 to 66.0%) in cockroaches on the fungal diet.

With regard to network structure, mismatched diet-microbiome pairs produced more modular networks, potentially due to more niche segregation and less overall interconnectivity (Fig. 5; see also Table S9 at https://www.doi.org/10.5281/zenodo.4074900). The communities thus appear more fragmented rather than unified in mismatched diet-microbiome pairs. Although we found a negative correlation between ASV abundance and its degree (*P* < 0.001 for all groups; see Fig. S11 at the Zenodo URL above), these patterns appear to in part be driven by ASVs belonging to differentially abundant genera. The most central node is always one such ASV (see Table S9 at the Zenodo URL above), and ASVs belonging to differentially abundant genera are more connected in the networks of their respective treatments (all but one *P* < 0.0001; see Fig. S12 at the Zenodo URL above). Differentially abundant taxa are thus both more abundant and more connected in their respective groups, and one could interpret them as leaver taxa, i.e., guiding microbial community structure to the different microbiome assemblages we observe (Fig. 2) (53).

### Functional prediction changes associated with changing inoculum and diets.

**FIG 6:**
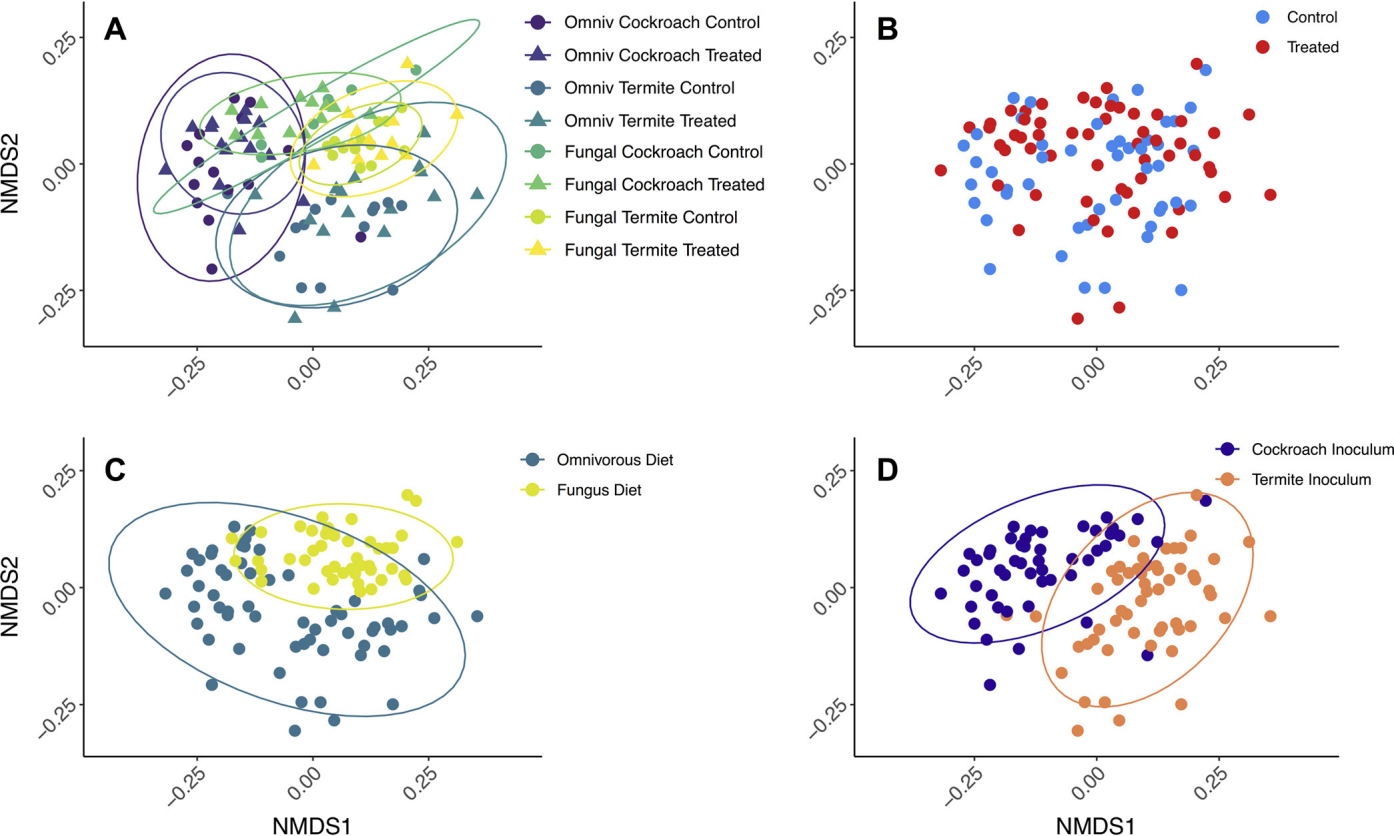
Bray-Curtis distance NMDS plot of the predicted bacterial metabolic pathways (PICRUSt2) of gut microbiomes of cockroaches with different gut inocula and diets, stress = 14.6%. (A) All groups; (B) antimicrobial treatment; (C) diet; (D) inoculum; coloration of treatment groups as in Fig. 2. Ellipses indicate 95% confidence intervals.

To discern any changes in predicted functional capabilities of bacterial communities as a whole, we employed PICRUSt2 on the 16S rRNA sequences (54) and subsequently identified predicted microbial metabolic pathways using the MetaCyc database (55). Overall, there were 406 predicted pathways in the cockroach gut microbiomes. Although microbial function (mean ± SD; Bray-Curtis distance: 0.1906 ± 0.079) was more conserved than microbiome composition (0.8604 ± 0126) (Fig. 6; see also Fig. S13 and Table S10 at https://www.doi.org/10.5281/zenodo.4074900), consistent with previous studies (56), there were clear differences in response to treatments (see Table S11 at the Zenodo URL above). Diet had a strong and significant impact on the functional profiles, explaining 22.2% of the observed variation (PERMANOVA_10,000 permutations_; F_1_ = 37.08, *P* = 1e−5) (Fig. 6C). Gut inoculum (F_1_ = 10.30, *P* = 5e−5) (Fig. 6D), antimicrobial treatment (F_1_ = 3.081, *P* = 0.0185) (Fig. 6B), and genetic background (F_10_ = 2.631, *P* = 0.0002) (see Fig. S14 at the Zenodo URL above) also had significant impacts, yet the variation explained by these factors was lower (6.2, 1.9, and 15.8%, respectively) (Fig. 6).

The microbiome responded to a fungal diet by increasing slightly MetaCyc pathway richness and diversity (WelchADF; WJ = 192.8, df = 1, *P* < 1e−10; WJ = 24.95, df = 1, *P* = 6.666e−6) (see Fig. S15A and S15B and Tables S12 and S13 at https://www.doi.org/10.5281/zenodo.4074900). Termite inoculum increased pathway diversity but not richness (WJ = 9.468, df = 1, *P* = 0.0033), while antimicrobial treatment increased pathway richness in hosts inoculated with cockroach guts (WJ = 5.115, df = 1, *P* = 0.0268). Subsequently, we analyzed functional divergence as we did for microbiome composition divergence (see Materials and Methods and above). Mirroring composition divergence, the omnivorous diet produced a more variable functional microbiome than the fungal diet (WJ = 12.42, df = 1, *P* = 0.0008) (see Fig. S15C and Table S14 at the Zenodo URL above). Interestingly, an interaction between diet and inoculum indicated that when these are matched (i.e., cockroach inoculum together with omnivorous diet and termite inoculum with fungal diet), there is significantly lower functional divergence than when they are mismatched (WJ = 8.251, df = 1, *P* = 0.0056).

**FIG 7:**
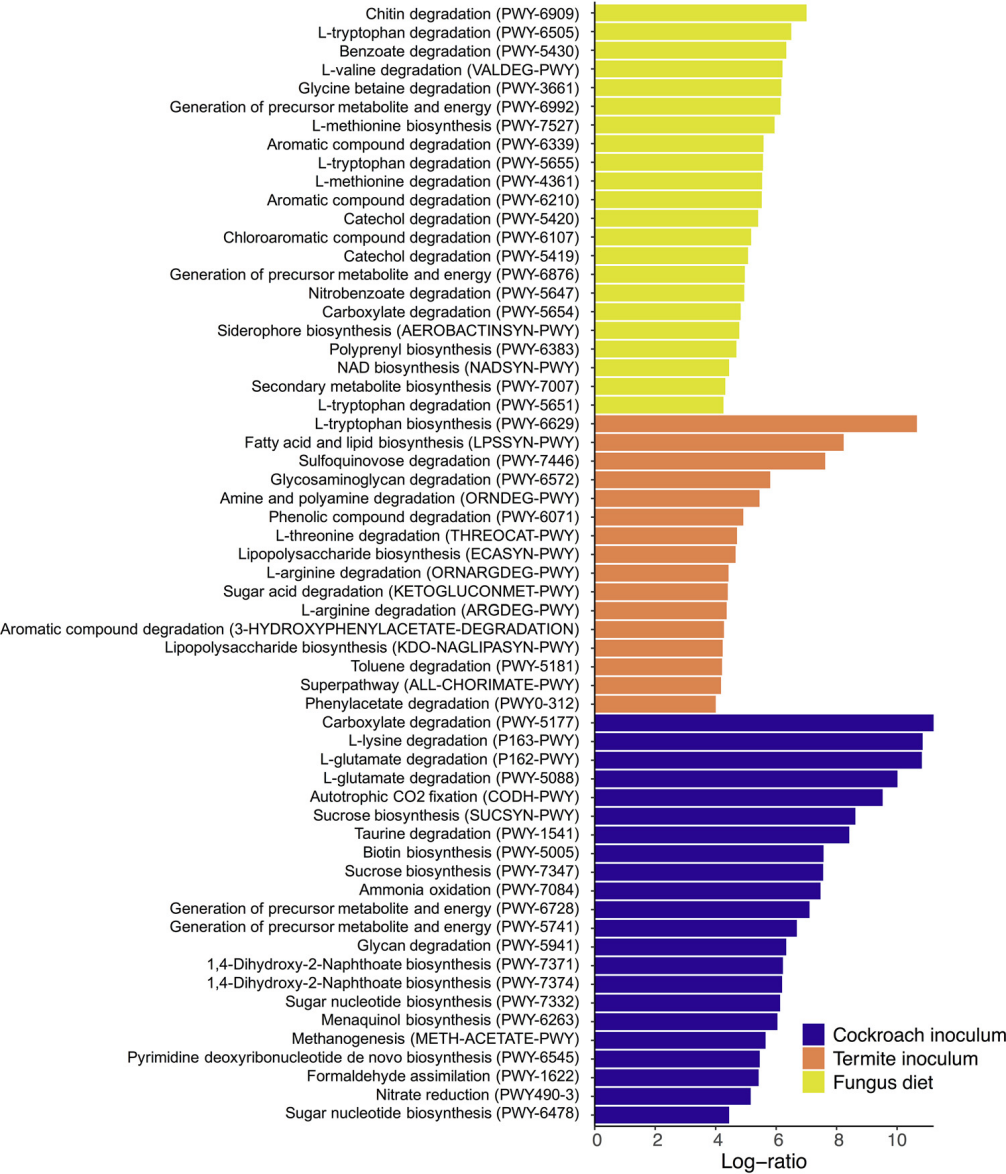
Log-ratio (effect size) increase in functional metabolic categories (pathways in parentheses) that significantly correlated with inoculum or diet compared to their alternatives, derived from a multivariate Aldex2 generalized linear mixed model. Only log-ratios above 4 are shown; see Fig. S16 at https://www.doi.org/10.5281/zenodo.4074900 for all significant pathways and Table S15 at the same Zenodo URL for the full results.

To test for differences in predicted pathway abundances between treatments, we ran Aldex2 analyses (17, 50, 51) (Table S15 at https://www.doi.org/10.5281/zenodo.4074900 gives the full results and Fig. S16 at the Zenodo URL above gives all significant pathways; Fig. 7 shows significant pathways that increased more than 4-fold in centered-log ratio abundance). A total of 266 pathways were significantly enriched across treatment groups, with 26 being in cockroaches on a fungus diet, 138 on the omnivorous diet, 70 with termite inoculum, and 57 with cockroach inoculum. Of these, only 30 overlapped between groups: 26 between cockroach inocula and omnivorous diet, one between cockroach inocula and fungal diet, one between termite inocula and omnivorous diet, one between termite inocula and fungal diet, and one between antimicrobial-treated cockroaches and the omnivorous diet (see Table S15 at the Zenodo URL above). Among pathways increasing in cockroaches on a fungal diet, 24 increased more than 4-fold, most prominently for chitin breakdown due to the abundance of this carbon source in the diet. Pathways increasing in abundance also included several amino acid degradation pathways (three out of four were for l-tryptophan), as well as a series of pathways for aromatic compound degradation (Fig. 7). The role of termite gut bacteria in the breakdown of lignin-derived aromatic compounds (57) likely caused seven pathways for aromatic compound degradation to increase more than 4-fold when cockroaches were offered a termite gut inoculum. Three pathways associated with proteinogenic amino acid degradation were also increased in this group. Predicted proteinogenic amino acid degradation also increased in cockroaches fed a cockroach inoculum, as did several pathways for sugar biosynthesis were predicted. Interestingly, the omnivorous diet did not lead to increases of any pathways more than 4-fold, although a high number of pathways did significantly increase in cockroaches on this diet (see Table S15 at the Zenodo URL above).

Finally, we tested whether any treatment enriched broader functional categories in differentially abundant MetaCyc pathways (see Table S16 at https://www.doi.org/10.5281/zenodo.4074900). Overall, the omnivorous diet treatment led to enrichment of biosynthesis pathways (Fisher’s exact test, p_adj_ = 3.748e−9), particularly the major categories of amino acid (p_adj_ = 1.945e−8) and nucleoside/nucleotide (p_adj_ = 2.276e−8) biosynthesis and the functional groups of proteinogenic amino acid (p_adj_ = 1.806e−5) and purine nucleotide biosynthesis (p_adj_ = 0.0436) and degradation (p_adj_ = 0.0002). In cockroaches on a fungal diet, however, degradation, utilization, and assimilation pathways were enriched (p_adj_ = 0.0449). The termite inoculum enriched phenolic compound degradation (p_adj_ = 0.0493) and fatty acid (p_adj_ = 0.0456) and quinol/quinone (p_adj_ = 0.0017) biosynthesis.

## DISCUSSION

*Shelfordella lateralis* cockroach hatchlings emerging from their ootheca rely on independently acquiring all bacterial inocula to colonize their guts, except for vertically transmitted *Blattabacterium* endosymbionts (cf. reference 48). Our findings suggest that the first microbial colonization is through bacteria present on the ootheca, implying an extent of vertical transmission. This impacts subsequent invasions by microbes, consistent with how early-life microbiota has a disproportionate impact on community assembly, in part due to priority effects allowing initial colonizers to take a strong hold (34, 35, 58, 59). We found this priority effect to only slightly affect which microbiome the cockroaches had a month later when the experiment was terminated. However, oothecal microbes in the gut did compete with subsequent colonizers, thus, guiding microbiome assembly toward a simpler community compared to that when ootheca microbes were depleted (Fig. 3A). Each colonizing microbe can stochastically lead community assembly in diverging directions and toward different stable states (cf. reference 59). The more stochastic this process is, the noisier the community assembly is expected to be. Competition offered by the very-early-life inoculum, and the consequent decrease in colonizing microbes, allows community assembly to be more reproducible (cf. the consistency patterns in Fig. 3B) as has been elegantly shown before in the simpler communities hosted by *Drosophila* fruit flies (59). This points to a stabilizing role of semi-vertically transmitted taxa in *S. lateralis* and, consequently, to the contribution of host traits enhancing initial inoculum toward ensuring that the microbiota develops reproducibly. This interpretation is in line with previous work elucidating the importance of competition for microbiome stability (60, 61), an effect which seems to start at the very earliest stages of microbial assembly in both simple (59) and complex (this study) host-associated microbial communities.

After 28 days of treatment, cockroach microbiomes shifted most prominently according to the single event of environmental microbial inoculum and, secondarily, according to the continuously provisioned diet. This led to four distinct clusters in NMDS space that correspond to the combination of diet and inoculum (Fig. 2A and see Fig. S6 to S9 at https://www.doi.org/10.5281/zenodo.4074900). The strong impact of inoculum implies that a single experimental introduction event with microbes able to colonize a gut system can lead to remarkably distinct microbiome structures. This underlines the impact that the gregarious lifestyle of *S. lateralis*, combined with coprophagous behavior (30), has by offering ample opportunities for microbial uptake from conspecifics. This is consistent with recent work showing that fecal pellets in the diet can restore microbiomes after antibiotic treatment of Blattella germanica cockroaches (44). Microbe-sharing benefits of group living (30, 62–65) can thus reliably ensure horizontal transfer of gut microbes from the environment and eliminate the need for vertical transmission for the acquisition of a taxon-specific microbiota. Such behavioral microbiota filtering can contribute to patterns of phylosymbiosis, i.e., matches between host phylogeny and microbial community composition (2, 65), and help explain codivergence between Blattodea and some of their microbial symbionts (66).

Host-symbiont adaptations are expected to allow conspecific inocula to provoke higher colonization rates than allospecific inocula, and this is what we observe. Cockroach inocula have more strains that are able to colonize guts than a related termite microbiome, despite hosting a markedly less diverse gut microbiome than the termite species (23). Consistent with this assertion, taxa that have diversified within Blattodea, and whose respective strains are shared by cockroaches and termites (36, 52, 66), display improved colonization success and increase in relative abundance when originating from a cockroach compared to a termite gut (Fig. 4), in support of host-symbiont adaptations (cf. reference 37). However, this increase in colonization rate by conspecific symbionts may lead to a decrease in microbiome consistency (Fig. 3B). Since more taxa are able to establish, and colonization is stochastic, a conspecific inoculum leads community assembly in more divergent directions and toward different stable states (cf. reference 59). The stabilizing role of semi-vertically transmitted bacteria is consequently also most prominent under a conspecific diet and inoculum, and it is absent with allospecific diet and inoculum (Fig. 3B). This further emphasizes the importance of host-microbe association specificity, insofar as their effects on ecosystem stability take place only while interacting with a host-specific community or under the influence of the diet that hosts have adapted to.

While microbial inoculum had the strongest impact on community structure, functional inference tells a different story: diet had by far the largest impact on predicted microbial metabolism, explaining over 3-fold more variation than inoculum (Fig. 6; see also Table S11 at https://www.doi.org/10.5281/zenodo.4074900). The predicted ability of the microbiome to digest diet compounds generally shifted from nitrogen-rich nutrients on the omnivorous diet to fatty acid and aromatic compounds on the fungal diet. While protein content is not markedly different between the two, the latter is consistent with the high fatty acid content of *Termitomyces* species (67). Several taxa increased in abundance in response to a fungus diet and may degrade by-products from lignin degradation by *Termitomyces* (e.g., *Enterobacteriaceae*, *Stenotrophomonas*, and *Pseudomonas*) (68–70, 114) (Fig. 4). The fact that diet enriched these taxa and functional groups, irrespective of which inoculum they received (Fig. 6 and 7), suggests substantial redundancy in cockroach and termite microbiome functions, a well-established phenomenon across even disparate microbiomes (56). Therefore, inoculum and diet play their roles at different levels: colonizations are largely driven by what microbes are present in the environment and their ability to establish within hosts, while microbiome function is largely driven by what diet this host subsequently receives.

Although PICRUSt2 analysis should be evaluated with caution as it is extrapolative from 16S rRNA gene sequences, we find plausible specific functional changes that are relevant for specific diets, such as chitin degradation in cockroaches on the fungal diet and differences in amino acid metabolism across diets (Fig. 6 and 7). Other predicted enrichments are more likely to be associated with the functions that community members have in their original host. For example, aromatic compound degradation among termite inocula is more likely to be significantly enriched because these metabolic capacities are important in fungus-growing termites, where gut microbes contribute to lignin decomposition at the early stages of plant biomass decomposition (57), than functionally relevant when cockroaches are sustained on pure-culture fungus biomass. Further elaboration beyond *in silico* predictions will be needed to identify genes in these pathways and explore their diet-associated expression.

Diet-imposed selective pressure on the microbiome resulted in two clearly differentiated microbiome structures (Fig. 2C and Fig. 4) and functional capabilities (Fig. 6C and Fig. 7). This flexibility is likely pivotal for the ability to track environmental changes and optimize nutrient intake or toxin degradation that maximizes host fitness (3, 14, 15). This is in line with ample evidence for animals filtering their microbial community through exposure to different environments (37, 71), including via social interaction in insects (72, 73) and primates (29, 74, 75) or more intricate mechanisms such as coprophagy in wood-feeding cockroaches (30) and trophallaxis in social insects (30, 76–79). The consistent patterns support that hosts are able to plastically uptake beneficial microorganisms, thereby enhancing host fitness through its extended phenotype—its microbiota—in an environment-specific way.

The omnivorous diet imposed a stronger filter on the cockroach microbiome, which ended up being less diverse than in cockroaches fed a fungal diet. While inoculations can cause both colonizations and extinctions (i.e., colonizers can outcompete residents), nutrient uptake can cause extinctions (i.e., if bacteria are outcompeted on a specific set of nutrients), and while microbes can enter with food, they will provoke fewer colonizations than inocula. This is backed by the fact we see that the strongest impact on microbiome composition is the single inoculation event, and, only a single genus was unique to the two omnivorous diet treatment groups while absent from all other groups (see Fig. S2 at https://www.doi.org/10.5281/zenodo.4074900). The omnivorous diet may have provoked more extinctions than the fungus diet, as it led to a less diverse microbiome (Fig. 3A). Such extinctions, occurring at a certain rate, can be considered stochastic processes which can lead the community in divergent ways, similarly to colonizations as discussed above (cf. reference 59). Therefore, microbiomes under the conspecific omnivorous diet, where more extinctions occur, showed more microbiome variability than those under a specialized fungal diet (Fig. 3B). Moreover, the fact that inoculations lead to microbial colonizations while diet mostly leads to extinctions may explain the inverse correlation between diversity and microbiome divergence between diets, as opposed to between inocula and between cockroaches with or without vertically transmitted taxa, in which microbiome divergence is linked to higher diversity (Fig. 3). Therefore, diets which sustain more diverse bacteria lead to more consistent microbiota, while inocula with more diverse bacterial colonizers lead to more divergent microbiome assemblies.

Our network analyses support that the higher microbiome variability in response to the omnivorous diet is unlikely to be a consequence of nutrient availability. One could predict that the omnivorous diet includes more universally available compounds and that the fungus diet includes more recalcitrant compounds. The latter would necessitate cooperation (positive interactions) between multiple specialized groups, while the former would lead to competition (negative interaction) for the universally available compounds. While this would be supported by the fungus diet leading to richer and more diverse functional microbiomes (see Fig. S15A and B and Tables S12 and S13 at https://www.doi.org/10.5281/zenodo.4074900), our networks, however, show that it is the omnivorous diet that leads to an ∼5% higher number of positive interactions, pointing away from a role of compound availability to explain microbial community stability (see Table S9 at the Zenodo URL above).

Inoculation events drive communities toward different stable states depending on colonizing or extinct taxa and ecological relations between the microbiota and hosts and other gut bacteria. Conspecific inoculation opportunities offer hosts and microbes the opportunity to spend prolonged amounts of time in each other’s presence, opening the door to coevolution (2), while diet shapes the functional capabilities that the microbiome houses. These processes are simultaneous, and highlight the importance of understanding microbial communities through both taxonomic and functional lenses (80–82), as ecology has done before microbiome science (83, 84). Although the relative roles of all potential factors shaping community structure will conceivably vary with hosts and diets, our findings suggest that environmental microbial sources have a stronger filtering effect on the microbiota composition than does diet, ecological relationships between early-life microbiota, or future gut colonizers. This, in turn, may lead to consistent microbial communities with host adaptations maximizing the extended phenotype that the microbiota provides (11, 85), particularly if microbes spend most of their lives in host-associated environments and, in turn, can coevolve with hosts (2).

## MATERIALS AND METHODS

### Study species.

Omnivorous *S. lateralis* reproduce sexually and cover their eggs in an ootheca, which is deposited and left until hatching (Fig. 1A). Adults do not provide parental care, and nymphs are self-sufficient after hatching and obtain microbes via coprophagy and selective recruitment from the environment (36, 86, 87). After obtaining an *S. lateralis* colony from an online breeder (https://www.ebay.com/usr/zoofoods), the colony was kept in a large plastic box (57 × 40 × 20 cm) with an aluminum mesh at 27°C and 50% relative humidity (RH). Cockroaches were fed *ad libitum* with dog food (Hund; Netto A/S, Denmark) with macronutrient composition of ca. 58% carbohydrates, 21% proteins, 10.5% fats, and 3% crude fiber. Water was supplied with soaked water-absorbing polymer crystals (http://insektorama.dk/koeb/vand-krystaller/). As an allospecific gut inoculum source, we used workers of the fungus-growing termite species *M. subhyalinus* (Termitidae, Macrotermitinae) from a laboratory colony at the Université Sorbonne Paris Nord. Termites were kept at 28°C and 75% RH, fed with dry wood, and kept humid with water-soaked paper towels.

### Antimicrobial (peracetic acid) treatment of cockroaches.

Twelve oothecae that produced between nine and 19 individuals were collected from the main colony. Six oothecae received an antimicrobial treatment by rinsing in 0.1% sodium dodecylbenzene sulfonate (Sigma-Aldrich, Germany; CAS number 25155-30-0), followed by 5 min in 2% peracetic acid (Supelco, Denmark; CAS number 79-21-0) and a rinse in 5 ml sterile water (Sigma-Aldrich, Germany; CAS number 7732-18-5) per the protocol of Tegtmeier et al. (47) and Mikaelyan et al. (37), who also did not find any apparent negative effects on cockroaches. Untreated oothecae were brushed free of dirt and were rinsed with 5 ml sterile water to mimic handling. Oothecae were subsequently placed individually in sterile 50-ml polypropylene Falcon tubes; cockroaches hatched from the ootheca after 27 ± 2 days (mean ± SE) (Fig. 1).

We assessed the effectiveness of the antimicrobial treatment using one cockroach per ootheca (Fig. 1; see also Table S17 at https://www.doi.org/10.5281/zenodo.4074900). A hatchling was crushed in 300 μl 0.2% saline solution (Sigma-Aldrich, Germany; CAS number 7732-18-5; Sigma-Aldrich, Germany; 7647-14-5) and vortexed, and 50 μl of the mixture was plated onto two different potato dextrose agar (PDA) plates (39 g/liter PDA, 10 g/liter agar). The remaining 200 μl was snap-frozen in liquid nitrogen and stored at −20°C for HiSeq amplicon sequencing (see below). One plate was kept anoxic in a GasPak 100 anaerobic system (Becton, Dickinson, USA), while the other was left at ambient oxygen levels. Plates were left at room temperature for a minimum of 1 month, the number of CFU was counted for each morphologically distinct microbe (up to 100 per unique morphology), and results were visualized in R (88) using the package ggplot (89).

### Diet manipulation and gut inoculum test.

After hatching, cockroach nymphs were starved for 5 days within the polymer tubes and in the presence of their ootheca of origin from which they hatched. Tubes were placed on ice for 15 min to ease handling of nymphs, prior to being individually placed into plastic boxes (7.5 × 5.5 × 5 cm) with lids that had holes covered by an aluminum mesh to allow ventilation, and a shelter made from an upside-down cardboard chicken egg-holder that was UV sterilized for 30 min prior to use. Nymphs from both antimicrobial-treated and control oothecae were distributed to one of the four treatment groups (Fig. 1). The number of nymphs used from each ootheca varied (see Table S17 at https://www.doi.org/10.5281/zenodo.4074900).

Nymphs were offered a single gut inoculum (cockroach or termite) in a feeding block as soon as they were moved to their experimental cages, which was removed a day later. To create feeding blocks, adult cockroaches and termite workers were placed on ice for 5 min and then euthanized by removing the head. Dissected guts (one gut per individual to be fed with the inoculum) were first mixed in 0.2% autoclaved saline solution and subsequently homogenized in 150 μl per gut in Potato dextrose broth (PDB; 39 g/liter PDB) by crushing and brief vortexing. The PDB/gut mixture was then mixed with gelatin (Sigma-Aldrich, Germany; CAS number 9000-70-8) to acquire solid feeding blocks, which were divided equally among the respective individual cages.

Respective diets were given *ad libitum* alongside the soaked water crystals. The fungal diet consisted of fresh mycelium of a *Termitomyces* species (the food source of the termite inoculum source) isolated from the fungus gardens of an *Odontotermes* cf. *badius* mound (termite colony code ICOO20) collected in Ivory Coast in 2018. Assuming that hyphal biomass is similar to mushrooms, a fungal diet would be comprised of dry weight biomass ranging from 43.7 to 57.4% carbohydrate, 15.1 to 19.1% protein, 2.5 to 5.4% lipid, and 17.5 to 24.7% crude fiber (67). The fungus was cultivated on PDA plates at room temperature until the mycelium covered the entire petri dish, after which it was harvested. Fungus-fed cockroaches had *Termitomyces* placed onto their water crystals to prevent the fungus from drying out. Individuals were fed twice per week, and leftover food was removed at each feeding session. After 4 weeks, the cockroaches were dissected, and individual guts were snap-frozen in liquid nitrogen and stored at −20°C until DNA extraction.

### Molecular methods.

DNA was extracted using the Qiagen DNeasy blood and tissue kit (Qiagen, Germany), following a modified version of the manufacturer’s protocol. Individual frozen guts (one gut, one sample) were crushed with a pestle that was subsequently rinsed with 180 µl ATL buffer. After adding glass beads, samples were vortexed for 15 s prior to adding 200 µl of chloroform-indole acetic acid (IAA). After centrifugation of samples at 20,000 × *g* for 15 min, 80 µl of the supernatant was transferred to a sterile Eppendorf tube and treated with 4 µl of RNase. The manufacturer’s instructions were followed during the rest of the extraction protocol. The volumes of ethanol and AL buffer were adjusted according to the digest volume (80 µl AL buffer and ethanol was added to the reaction mixture). Finally, samples were eluted twice using 100 µl of AE buffer to maximize DNA yield.

PCR amplification of the V3-V4 region of the 16S rRNA gene using the primer set 341F-806R (5′-ACTCCTACGGGAGGCAGCAG-3′; 5′-GGACTACHVGGGTWTCTAAT-3′) (90, 91). Initial PCRs were conducted to assess the presence of bacterial DNA. The PCR mixture (10 µl) contained 1 µl template, 0.4 µl of each primer, and 5 µl VWR Red *Taq* DNA polymerase master mix (VWR Chemicals, Denmark; CAS number 733-2547). The PCR conditions for amplification of DNA were as follows: initial denaturation at 94°C for 4 min, followed by 40 cycles of denaturation at 94°C for 30 s, annealing at 55°C for 30 s, and extension at 72°C for 30 s, and finally ending with 72°C for 4 min. Positive PCR was evaluated on a 2% agarose gel. Library preparation and PE300 HiSeq 2500 amplicon sequencing of 123 samples were done at BGI (https://www.bgi.com/us/), and all were successful, except the blank. Samples were sequenced in two batches: the first batch included guts from the preexperiment to evaluate antimicrobial effect, a mock community, and negative controls; the second included guts from the main experiment.

### Bioinformatics and statistical analyses.

Batches were handled identically using the dada2 pipeline v.1.12.1 (92) using RStudio v.3.6.1 (88). Default parameters were used aside from truncation length (275, 260), maxEE (2, 3), and minOverlap set to 20. Taxonomic assignment was first performed with the manually curated Dictyoptera-optimized Dict_db v.3.0 database (36), followed by reclassification using SILVA release 132 (93) of ASVs that were not classified to the genus level in the Dict_db v.3.0 database. This procedure allowed 96.4% of ASVs and 99.7% of reads to be classified to genus level in batch 1 and 92.1% of ASVs and 98.4% of reads to be classified to genus level in batch 2. *Blattabacterium*, an intracellular symbiont (48), was found in all negative controls, and these were therefore ignored, so taxa present in DNA-extraction kits have not been successfully removed. However, a cellular mock community standard (Zymobiomics; Nordic BioSite ApS, Copenhagen, Denmark) was used to control for extraction, PCR, and sequencing biases. All eight expected taxa were detected and classified appropriately, and no contaminants were detected in the mock community, despite lower DNA concentrations than biological samples, strongly suggesting sample cleanliness. dada2 removes singletons, and we filtered *Blattabacterium* and eukaryote ASVs, as well as ASVs with less than 10 observations in the full data set. Rarefaction plots (plotted prior to rare ASV removal but after dada2 removal of singletons; see Fig. S17 at https://www.doi.org/10.5281/zenodo.4074900) indicate that we captured a satisfactory portion of the full bacterial community. Alpha diversity was calculated on unrarefied ASV-level data using the R package phyloseq *estimate_richness* function v.1.28.0 (94); most results show Inverse Simpson, which is biased to abundant taxa. ANOVAs (95) were performed to calculate significant differences between experimental setups using the stats package v.3.6.1 (88), after testing deviation from assumptions with *shapiro.test* (96) and *bptest* (97) from the lmtest package v.0.9.37 (98). Whenever ANOVA assumptions were not met, a Kruskal-Wallis test was performed for univariate tests (99), or a Welch approximate degrees of freedom test for multivariate tests with a Games-Howell *post hoc* test was performed (100, 101). To assess community-level differences in gut microbiomes under different treatments, a permutational multivariate analysis of variance (PERMANOVA [102]) was performed on unrarefied ASV-level data using Bray-Curtis (103) distances with 10^4^ permutations using the adonis function from vegan v.2.5.6 (104). Alpha and beta diversity statistics were repeated on rarefied data yielding similar results (see Tables S4 and S6 at the Zenodo URL above). All adjusted *P* values were calculated with Benjamini-Hochberg correction (105). Community-level differences were plotted using a combination of dimension reduction analyses (nonmetric multidimensional scaling [NMDS; Fig. 2 and 6] and PCoA [see Fig. S6, S8, and S9 at the Zenodo URL above]) and distance metrics (weighted Bray-Curtis [Fig. 2 and 6; see also Fig. S9 at the Zenodo URL above]; unweighted Bray-Curtis, i.e., Jaccard [see Fig. S6 at the Zenodo URL above]; and UniFrac [see Fig. S7 and S8 at the Zenodo URL above]), using ggplot2 (89) and viridis (106). Finally, we calculated within-group microbiome consistency by inferring a per-group representative sample with the median abundance of each ASV from that group. Next, we calculated beta diversity between each sample in a group and the representative sample in that group (as in reference 49). Nonparametric WelchADF tests were used to test for significant differences between diets and between inocula and between antimicrobial-treated groups and controls since data significantly differed from homoscedasticity.

Differentially abundant bacterial genera between different treatment groups were identified following CoDa good practices (17, 50, 51) (https://github.com/ggloor/CoDa_microbiome_tutorial). A center-log-ratio-transformed multivariate model was conducted using the Aldex2 package (107) including the variables inoculum, diet, and antimicrobial treatment. After inspecting MA (log ratio − mean average) and volcano plots, significant values (p_adj_ < 1e−5) were extracted and plotted against their centered log ratio. As binning by genus is dependent on the order of classification database used, we repeated the analysis using SILVA first and Dict_db on genera unclassified with the former, as described above after switching the order of the databases (see Fig. S18 at https://www.doi.org/10.5281/zenodo.4074900). Functional prediction changes associated with different inocula and diets were evaluated across treatment groups using PICRUSt2 on 16S rRNA sequences (54). Subsequently, predicted microbial metabolic pathways were identified using the MetaCyc database (55) and analyzed statistically as the 16S rRNA data set for alpha diversity, beta diversity, and the Aldex2 model, always at the pathway level. Enrichment tests were performed with fisher.test using the stats package v.3.6.1 (88) and corrected with Benjamini-Hochberg correction (105).

For comparison with the inoculated communities, we sought available data on *Shelfordella lateralis* (36; *n* = 2) and *Macrotermes subhyalinus* (45; *n* = 1, worker) adults. We dereplicated and assigned taxonomy using Dict_db (36) and SILVA (93) as described above. Next, we merged phyloseq objects and clustered at the genus level to allow comparisons across sequencing platforms. Upset plot was generated with the UpSetR package v1.4.0 (108) (also see Fig. S2 at https://www.doi.org/10.5281/zenodo.4074900).

To better elucidate the associations between ASVs with more than 500 reads in each of the four diet-inoculum treatments, we generated microbial networks (Fig. 5), using the Spiec.Easi package v.1.1.0 (109) with default parameters and the “mb” algorithm and in line with good practices for microbiome networks (53). Lambda optimization was performed to obtain networks with stability closer to the 0.05 threshold, recommended by the authors of the package (109). Networks were analyzed with ggnet v.0.1.0 (110), igraph v.1.2.5 (111), network v.1.16.0 (112), and ggnetwork v.0.5.8 (113).

### Data availability.

HiSeq data are available from the SRA archive at NCBI (BioProject PRJNA642018). All scripts used are included as supplementary material at https://www.doi.org/10.5281/zenodo.4074900.
